# Food Addiction and Binge Eating: Lessons Learned from Animal Models

**DOI:** 10.3390/nu10010071

**Published:** 2018-01-11

**Authors:** Marta G. Novelle, Carlos Diéguez

**Affiliations:** Department of Physiology, Center for Research in Molecular Medicine and Chronic Diseases (CIMUS), University of Santiago de Compostela-Instituto de Investigación Sanitaria (IDIS), CIBER Fisiopatología de la Obesidad y Nutrición (CIBERobn), Instituto de Salud Carlos III, 15786 Santiago de Compostela, Spain

**Keywords:** eating addiction, opioids, dopamine, obesity, binge eating, animal models

## Abstract

The feeding process is required for basic life, influenced by environment cues and tightly regulated according to demands of the internal milieu by regulatory brain circuits. Although eating behaviour cannot be considered “addictive” under normal circumstances, people can become “addicted” to this behaviour, similarly to how some people are addicted to drugs. The symptoms, cravings and causes of “eating addiction” are remarkably similar to those experienced by drug addicts, and both drug-seeking behaviour as eating addiction share the same neural pathways. However, while the drug addiction process has been highly characterised, eating addiction is a nascent field. In fact, there is still a great controversy over the concept of “food addiction”. This review aims to summarize the most relevant animal models of “eating addictive behaviour”, emphasising binge eating disorder, that could help us to understand the neurobiological mechanisms hidden under this behaviour, and to improve the psychotherapy and pharmacological treatment in patients suffering from these pathologies.

## 1. Introduction

Eating disorders (ED), defined as disturbances in eating habits characterised by insufficient or excessive food intake causing energy imbalance, are associated with high comorbidity and have serious health consequences. Therefore, although the prevalence for ED has remained stable, the high mortality rate, the association with other psychiatric disorders, and an increased level of awareness of eating disorders between the general population and clinicians have encouraged researchers to investigate the genetic, neurochemical, and physiological substrates implicated in ED [1,2]. In a highly obesogenic environment, much attention has been given to ED characterised by compulsivity and overeating, including binge eating disorder (BED), certain forms of obesity, and the newly proposed construct of “food eating addiction” [3]. Throughout this review we will briefly cover current knowledge of the neurobiology of feeding behaviour, focusing on non-homeostatic circuits, and we will look over the controversy about the misconception of “food addiction”. Finally, we will explore new evidences learned from animal models in order to get a better understanding of BED, recently integrated as a novel diagnosis into the Diagnostic and Statistical Manual of Mental Disorders (DSM-5).

## 2. Understanding the Neurobiology of Eating Behaviour. Eating beyond Metabolic Needs

Food intake is an essential behaviour for survival and highly regulated by homeostatic, hedonic and learned cues. Consequently, eating behaviour depends on a simultaneous functioning of homeostatic pathway together with a more flexible non-homeostatic one, whose functions can vary between individuals according to previous experiences and/or epigenetic variations [4,5,6,7,8].

New insights argue that the impact of the modern food environment is mainly on cortico-limbic brain systems dealing with reward, emotion and cognition. Signals from the cognitive and rewarding brain may override classic homeostatic regulation leading to development of obesity or eating disorders. These stimuli follow pathways that include, but are not limited to, corticolimbic regions within the amygdala, hippocampus, and thalamus; mesostriatal dopamine (DA)-gated circuits within the nucleus accumbens (NAc) and the ventral tegmental area (VTA); and prefrontal cortex (PFC) regions predominantly within the orbitofrontal projections [9,10,11,12].

In this context, Berridge and collaborators described three aspects of reward: liking, wanting, and learning, that despite being tightly linked they can be dissociable in terms of their neural substrates yet. So, while liking and wanting, respectively, refer to the hedonic impact of and the motivation for a reward, the learning process comprises the associations with and predictions about rewards [13,14]. Animal models have mainly associated opioid, cannabinoid, orexin and γ-aminobutyric acid (GABA) systems as mediators in the “liking” experience, via coordinated activity in a network of hedonic hotspots in the nucleus accumbens, ventral pallidum and brainstem. Moreover, these neurotransmitters can be also implicated in other processes of the reward regulation, as opioids enhancing the “wanting” [15]. On the other hand, the mesolimbic dopamine system is crucial in the “wanting” and “learning” components [13,16]. It should be pointed out that “liking” and “wanting” systems are essentially pure “go” systems. That means, once they are activated cannot be diminished by satiety influences, they never generate a strong “stop” signal to halt intake, they only tone down the intensity of the “go” [17]. Interestingly, the incentive sensitization theory of addiction proposed by Robinson and Berridge, is based on a pathological incentive motivation (wanting) for drugs even after the discontinuation of drug use, that can be manifest in behaviour via either implicit (as unconscious wanting) or explicit (as conscious craving) processes, depending on circumstances. These features are linked with learning mechanisms that normally direct motivation to specific and appropriate targets [18,19,20]. Likewise, excessive “wanting” and “liking” for food, notably hyper-palatable food, may play a role in overeating. Moreover, as in in drug dependence, the attractive and rewarding properties of hyper-palatable foods do not remain confined to the reward itself. Reward-related cues, in this case food cues, can be attributed with excessive incentive salience and become signals that draw attention and trigger overconsumption [21]. In other words, the wanting of addiction is connected less with pleasure (liking foods), and more with the negative reinforcement created by their withdrawal. Consequently, wanting and craving high sugar and high sugar/high fat foods are more about trying to prevent the return of negative feelings [22,23].

### 2.1. The Role of Opioid System, More Than “Liking” Regulation

‘Liking’ and ‘disliking’ of a food was determined by carefully observing the orofacial expressions of rats drinking caloric test solutions. The original idea to observe rats’ facial expressions to measure how much pleasure (or aversion) they are getting from a given food was inspired by earlier human studies and later adapted for rodents [24,25,26].

As we already have commented above, endogenous opioid system is crucial in “liking” aspect of the reward process. Despite of opioids are involved in a broadly distributed neural network, affecting both homeostatic and hedonic mechanisms; the dominant view is that opioids, especially the mu-opioid system, regulate the “hedonics of feeding” by their modulation of the palatability of food regardless of the caloric value presented. Opioids stimulated ingestion of “attractive” diets in sated rats, enhanced the “dessert effect” when a palatable food was offered at the end of a regular meal [27]. On the other hand, opioid antagonists attenuate appetite for palatable food. Thus, craving for palatable food could be considered as a form of opioid-related addiction [28,29].

All these conclusions are largely based on evidence obtained from animal models. Some studies tried to elucidate if opioids stimulate intake of specific macronutrients or of preferred foods. Several works showed that when rats could select the macro composition of the diets, after an injection of morphine, a µ-opioid receptor agonist, animals had higher preference for fat intake, instead of carbohydrate intake [30], in contrast, the opioid antagonist, naloxone preferentially decreased fat intake [31]. According to these data, opioids regulate the intake of specific macronutrients. However, when the baseline dietary preferences of the rats were considered, after morphine injections, it was observed that opioidergic modulation of feeding may be driven more by individual preference than by macronutrient, since morphine primarily stimulated carbohydrate intake in the carbohydrate-preferers, and stimulated fat intake in the fat-preferers [32]. Glass et al. reported a complementary result with naloxone injections: the intake of the preferred diet was reduced by naloxone at lower doses than those required to reduce intake of the less-preferred diet [33]. Lately, it was observed that the opioid effects of preferred versus non-preferred food were dependent on the site of injection. Therefore, while the naltrexone (NTX, preferential μ-opioid receptor antagonist) injections in the central nucleus of the amygdala caused a decrease in intake of the preferred food, injections in the paraventricular nucleus of hypothalamus caused a decrease in the intake of both foods [34]. The role of µ-opioid stimulation is not only region-dependent. While µ-opioid stimulation via microinjection of DAMGO (µ-agonist) within the rostrodorsal quadrant of NAc medial shell can double the hedonic impact of sweet tastes, stimulation in other sub regions of medial shell does not increase “liking” reactions to sweet food [35,36]. Finally, it should be noted that hedonic enhancement is also receptor (mu, delta, kappa) dependent [37]; although μ- and κ-opioid receptor stimulation increased the ‘liking’, only the μ-opioid receptor stimulation increased the incentive motivation for food [38]. Other researchers also provided some evidence of the role of opioid system either macronutrient or preference-specific effects on food intake. These studies showed that nucleus accumbens opioidergic effects were influenced by the relative preference for specific foods, foods high in fat or sugar. When both high-fat and high-sugar foods were available simultaneously, opioid stimulation increased intake more for the high-fat food [39,40,41]. However, Mena and collaborators showed that intra-PFC μ-receptor stimulation augments the reward valuation of carbohydrate-enriched foods, along with several behavioural changes such as a high-arousal and stress-like state. The authors suggest that this carbohydrate hyperphagia could represent an attempt to suppress a stress-like aversive state, since PFC is significantly activated by stress [42]. One mechanism that could explained the opioid-mediated overconsumption of palatable foods is through delaying the satiety systems; either the melanocortin or oxytocin systems [27]. These preferences and craving for appetizing foods were also observed in several human studies [43,44,45,46].

Beyond “liking” process, opioid stimulation directly causes increased cue-triggered ‘wanting’ as well as dopamine stimulation. Therefore, opioid mechanisms can also regulate incentive motivational, to wit propensity to seek palatable foods [37,38,47]. In fact, for example, the injection of non-selective opioid receptor antagonist, nalmefene, blocked the anticipatory negative contrast in the binge-eating procedure as well as highly palatable food binge eating [48]. External food-related cues, learned cues, can also precipitate the desire for food, increasing the food craving independently of homeostatic needs. In this context, using Pavlovian-instrumental transfer (PIT) paradigm, that can model mechanisms responsible for producing “cue-triggered wanting” or craving [49], it was shown that opioid stimulation caused “wanting” as well as dopamine [47]. Additionally, NTX, and GSK1521498 (μ-opioid receptor antagonist) were tested on food seeking behaviour using chocolate-flavoured pellet reinforcement. Both compounds reduced food intake, but only GSK1521498 reduced the seeking responses for chocolate before ingestion, suggesting that μ-opioid system has a crucial role on incentive motivational mechanisms controlling food seeking [50]. Noteworthy, the same compound also reduced motivational responding in binge-eating obese people in a placebo-controlled trial, although subjective liking increased following drug treatment [51]. It has been hypothesized that μ-opioid receptors localized on the GABAergic interneurons in the VTA may act to decrease dopamine release in the NAc to reduce food seeking and incentive motivation for food [38,52].

Additional studies have also confirmed the role of opioid systems in other brain structures in the motivational mechanisms underlying eating behaviour. Therefore, when central amygdala (CeA) was stimulated by DAMGO infusions (µ-opioid agonist) caused elevated incentive motivation in subjects naturally attracted both by a predictive cue (sign-trackers) and by a reward contiguous goal cue (goal-trackers); also, an increase in “wanting” behaviour under PIT model [53,54]. On the other hand, likewise, μ-opioid receptors within the medial prefrontal cortex (mPFC) mediate an important function in overeating. In fact, naltrexone microinfused into the mPFC selectively reduced the consumption and the motivation to obtain highly palatable food, but not standard chow [55], while intra-PFC DAMGO engendered “high-drive-like” effects [56]. In this context, Baldo group has suggested that neuroadaptations of the opioid system in mPFC could explain partly the development of binge-like eating [42,57,58]. Such as it has been recently demonstrated, mPFC exerts top-down control over midbrain dopaminergic interactions with the striatum and an increase in the mPFC can suppress natural reward-related behaviour [59]. Finally, it has been also reported that δ-opioid receptors in the NAc-Shell are involved in the effects of predictive learning on choice between actions. In fact, under a PIT paradigm, the treatment with δ-opioid receptor antagonist naltrindole, blocked this behaviour [60,61].

### 2.2. Dopamine System, the “Want” Pathway

The association between dopamine and food intake appears to date from ancient times and it has been postulated to be linked to and play a key role in human evolution. Specifically, it was proposed that that increased levels of dopamine were part of a general physiological adaptation of our ancestors due to an increased consumption of meat around two million years ago and later enhanced by further changes in macronutrient intake about 80,000 years ago [62]. At present there are clear evidences that dopamine pathways and dopamine receptors are involved in energy homeostasis. In fact, there are clear evidences linking all the five subtypes dopamine-receptors to energy balance and metabolic homeostasis. In this regard the FDA have approved a D2-agonist, bromocriptine, as adjunctive treatment for type 2 diabetes [63]. Moreover, most of all the FDA-approved antiobesity drugs, including liraglutide, appears to act largely through dopaminergic pathways [64]. On the contrary chronic consumption of dopamine antagonists as in patients with schizophrenia leads to enhanced eating and weight gain. In general, the relevance of dopamine in the integrated control of the homeostatic pathways involved in food intake is beyond any doubt. Detailed studies on dopamine involvement in leptin- and ghrelin-elicited changes in food intake are well established [65]. This dopamine effects appear to be mediated by both D1R and D2R, being the latest one the most relevant. Moreover, heteromers of GHSR1:DRD2 have been implicated in obsessive eating associated to Prader-Willi Syndrome [66].

Another property inherently relevant to feeding behaviour is the concept regarding to reinforcement, motivation and incentive salience [67,68,69,70]. While “liking” is closer to sensory processes, “wanting” is closer to decision making and motor action, by reflecting the cue-driven inclination to choose one behaviour over another to optimize reward. Dopaminergic projections from the VTA to the NAc and prefrontal cortex are the most important component of the implicit or unconscious “wanting” system [71]. Part of dopamine hypothesis of reward is based on the initial work conducted by Wise and collaborators, where animals subjected to DA antagonist pimozide (specially D2 receptor) showed a decrease in self-stimulation in ways that implied a devaluation of reward, a decrease in the pleasure of the reinforcer [72,73]. Through the years many other groups have arrived at same conclusions, DA is required for normal motivation and reward, and has a crucial role in feeding behaviour; in fact, animals lacking dopamine throughout the brain and body do not eat [74,75], although as it has been confirmed its role is brain-region dependent.

The NAc is a brain region in the ventral striatum that appears to play a crucial role in behaviours related to natural reinforcers and incentive as well as initiating key intracellular plasticity mechanisms required for learning about food resources [76,77]. Moreover, dopamine dynamics differ substantially between the NAc core and NAc shell in relation to distinct aspects of appetitive and aversive motivational states [78]. Pharmacological blockade of D1 and D2 dopamine receptors in the NAc affects motor conduct and has small effects on feeding patterns, but does not reduce the amount of food consumed. These effects can be interpreted as reflecting a more selective role for dopamine transmission in the anticipatory/approach phase versus the consummatory phase of feeding [79]. According to this idea, Salamone and colleagues carried out several interesting studies examining in deep the behavioural effects of moderate NAc dopamine depletions. They found that dopamine depletion reduced the motor effort to obtain food reward, but approach or intake did not decrease when food was clearly available [80,81,82], and still animals can have hedonic responses for food in the absence of dopamine [83]. Therefore, dopamine system would be responsive to reward predictors and seemingly unresponsive to the reward “itself” [73,84]. Nevertheless, restoring dopamine signalling selectively to the dorsal striatum, composed of the caudate and putamen, is sufficient to allow feeding, locomotion, and reward-based learning [85]. Besides, an increase of D2 receptors in the striatum are correlated with an optimal goal-directed behaviours and motivation [86,87].

In this context, the downregulation of striatal dopamine D2 function has been proposed to explain the reward deficiency or reward hyposensitivity theory [88]. According to this, a reduced D2R expression in the striatum, observed both in human and animal models, is a neuroadaptive response in order to compensate the overconsumption of palatable foods [89,90,91,92]. Furthermore, this reduced sensitivity potentially could predict a cause of excessive eating and/or obesity [89,90,92,93]. Consistent with the reward deficiency theory, some studies reported obese versus lean adults show lower striatal DA D2-like receptor availability [94,95], moreover obese adults have less capacity of nigrostriatal neurons to synthesize DA [96] and less striatal responsivity to tastes of high-fat/sugar beverages [97]. Also, patients with BED tend to have reduced level of DA in the brain [98]. Contrarily, other groups have shown higher striatal DA in obese individuals [77,99]. Despite some discrepancies regarding to striatal dopaminergic levels, the A1 allele of the D2/ANKK1 Taq1 polymorphism has been correlated with reduced D2R availability in the striatum, obesity and compulsive behaviour [77,100]. Likewise, D2 receptors were reported to provide a target for ameliorating binge eating behaviour in a rat model after NAc deep brain stimulation [101,102]. Therefore, while DA receptor 2 antagonism in NAc increases binge-like feeding [101], activation of serotonin 2C receptors in DA neurons inhibits this binge behaviour in mice [103].

It is important to note that in addition to its role in motivational processes, cortico-mesolimbic dopamine pathway also play an important role in mediating learning [14,104,105]. Changes in learning pathways might change rewarded responses. Learning processes are highly influenced by emotional and motivational components and required for reward prediction, for making anticipatory responses, for guidance by cues, and for goal-directed action. These cues from the environment, such as the sight and smell of food, or even advertisements for food, are learned and become associated with future reward [11,14]. A range of animal studies has demonstrated that food-associated cues can promote eating in the absence of metabolic requirements [106,107]. Thus, food-predictive cues can stimulate eating in adults and children, even when they are full [6,108]. The basis of cue-potentiated feeding (CPF) behaviour is Pavlovian conditioning. A feature of CPF is that it tends to be specific for the cued food and does not increase intake generally, which has similarities with the “cravings” experienced by binge eaters [109]. Recent studies have shown that dopamine has a selective role in stimulus–reward learning that is specifically associated with the attribution of incentive salience to reward cues. This fact could explain as individuals who attribute reward cues with incentive salience find it more difficult to resist such cues, a feature associated with reduced impulse control [110].

The mPFC receives information about cues in the environment via the sensory cortices, but also about the internal motivation to eat via dopamine neurones of the VTA [56,58]. A group of mPFC neurons, which contain the dopamine D1R receptors, are activated during hunger-induced food intake, and their stimulation and inhibition, increases and reduces feeding respectively. The main target of the D1R-containing subset of mPFC neurones is the medial BLA [111]. On the other hand, it has been hypothesised that the transition from voluntary drug use to more habitual and compulsive drug use represents a transition at the neural level from PFC to striatum; and a progression in the striatum from ventral to more dorsal domains, involving its dopaminergic innervation [112]. Moreover, reduced dopaminergic modulation has been suggested to impair inhibitory control over food intake and to increase risk of overeating in humans [95].

### 2.3. Are There Other Neurotransmitters or Hormones That Can Modify “Liking” and/or “Wanting” Behaviours?

In addition to the endogenous dopamine and opioid systems, various hormonal and neuropeptide systems influence performance in one or more of the food motivation behavioural paradigms described earlier. Signals such as leptin, insulin, ghrelin, glucagon-like peptide-1 (GLP-1) and melanin concentrating-hormone (MCH), orexins, oxytocin, serotonin between others, are involved in hunger and satiety signalling as well as reward-related neurocircuitry. The review of this topic is complex and beyond the aim of this paper, but detailed reviews of this topic can be found elsewhere [113,114,115,116,117,118,119,120,121,122,123].

## 3. Can We Talk about “Food Addiction”?

The concept of food addiction has been suggested for the first time by Randolph in 1956 [124], but only recently it has received due attention, mainly because of its correlation to the increasing rate of obesity. “Addiction is defined as a chronic, relapsing brain disease that is characterised by compulsive drug seeking and use, regardless of unhealthy consequences” [125]. This chronic relapsing disorder is comprised of three steps: preoccupation/anticipation (craving), binge/intoxication, and withdrawal/negative effect. These three stages interact with each other, becoming more intense, and eventually leading to the pathological state known as addiction. Not all drugs produce the same pattern of addiction, but lately the progression of this behaviour triggers alterations in normal brain function and consequently induces neuroplasticity in all the structures implicated [22,23,126,127]. Remarkably, addiction induces neuronal changes in prefrontal cortical and basal ganglia activities, leading to reductions in control and decision-making skills, and causes a chronic perturbation in brain reward homeostasis mainly in the mesolimbic dopamine system. Moreover, the opioid, GABAergic and glutamatergic neurocircuitries play a key role in the development of addiction [92,128,129].

In the context of environments saturated with food, where clearly, obesity has become a worldwide problem in a short time, and the binge eating disease is the eating disorder with more incidence [130,131] several questions have been put forward. Can overeating become a pathologic attachment to food? If so, can clinicians and researchers assert that food addiction is a new category of psychiatric disorder or brain disease? Compulsive sexual behaviour, pathologic gambling, and hedonic overeating are important problems, but are they addictions?

Although in some cases excessive consumption of food can fit with all DSM-required criteria according to some, food addiction was not included in newest edition of the of the DSM manual [132]. Although it can share some symptoms with BED and obesity, and includes behavioural patterns similarly to substance use disorders [129]. One fundamental distinction between currently accepted addictive substances and food is the fact that food is necessary for survival. Notably, there is a still an ongoing debate between the scientific community about whether food addiction is a misnomer and the phenomenon could be more accurately categorised by an alternative designation [92,129,133,134,135,136,137,138,139,140,141]. Therefore, while some researchers see overeating as substance use disorders, where people are addicted to sugar, salt, additives and high fat content [140,142,143]; other suggest increased food intake related to obesity or eating disorders should be considered as a behavioural addiction [136,144,145]. Different views on this topic are almost unavoidable if we accept that it is quite unlikely that any animal model of food addiction can mimicked to a large extent food/eating addiction in humans. However, some researchers have tried to study the food addiction in animals by following the three-criteria model proposed by Deroche-Gamonet et al. [146]. Tolerance, reduction in the effect of a drug resulting from of repeated exposure to the substance, was observed after extended access to a palatable diet [147] and also some data suggest that there is a cross-tolerance between sweet solutions and opioids [148]. In this context, Woods hypothesizes humans must learn to tolerate the intake of food in order to minimize its impact on the body, as they learn responses to help them tolerate the administration of dangerous drugs [149]. Regarding to the withdrawal component of addiction, negative effects after the abrupt discontinuation or decrease in intake of drugs, conflicting results have been reported depending on the kind of food. This is discussed below in Section 4.1.3. Many other aspects related to “food addition” concept have been studied in animal models too. By using a so-called time-out model Ghizta and colleagues have attempted to elucidate the difficulty to limit intake or food seeking; after prolonged training, animals exposed to palatable diet increase their food seeking responses [150]. Moreover, some studies have demonstrated that animals continue to seek the food despite adverse consequences [89,151].

For the first time, the DSM-5 grouped a disorder not involving substance use (gambling disorder) together with substance use disorders in a new category entitled: “Substance Related and Addictive Disorders” [132]. In this context, our group agreed to other researchers, and based on the current studies, consider that there is no enough evidence to conclude that a specific food, food ingredient or food additive can be addictive. Although is too early to draw definitive conclusions regarding the “food addiction” concept and further work is necessary, we consider that we should talk about “eating addiction” or more precise “addictive eating behaviour” [136,152]. In agreement with other authors [153], we believe that some animal models from drug addiction research, as the three-criteria model [146], should be applied in eating disorder field. These models may provide us new neuronal mechanisms in order to elucidate differences between “food addiction” and other compulsive eating behaviours.

## 4. Binge Eating Disorder, a “Full-Fledged” Pathology

The newest edition of the Diagnostic and Statistical Manual of Mental Disorders (DSM-5) has introduced important changes in the diagnostic system for eating disorders; trying to improve the ability for clinicians to arrive at a precise diagnosis [132]. Perhaps the most significant improvement with the DSM-5 is that Binge Eating Disorder (BED) is now considered as proper diagnosis in parallel to other main eating disorders as Anorexia Nervosa and Bulimia Nervosa [154]. BED is the most prevalent eating disorder (between 2–5% of the adult population) and more common in women than men. BED is characterised by compulsive episodes of disproportionate consumption of highly palatable foods together with a strong sense of loss of control. Binge-eating episodes are often accompanied by feelings of anxiety, shame, disgust and guilt, high risk of suicide, but they are not followed by compensatory purging behaviours. Although BED is often associated with obesity many BED patients have normal body weights [130]. Based on these characteristics this kind of behaviour could be described as an addiction-like behaviour; that is, an “eating addiction” [136]. However, despite of existing research suggests that there is an overlap between BED and the “eating addiction” neither all people with BED meet Yale Food Addiction Scale (YFAS) criteria for this addiction nor all people with eating addiction meet criteria for BED [152,155,156]. In this context, Hoebel’s group has modelled in rodents all the aspects of food addiction; “Bingeing”, “withdrawal”, “craving” and “cross-sensitization” with drugs of abuse. These researches have reported that sugar has addictive properties similar to psychostimulants and opioids, and confirmed it is possible to talk about sugar addiction as a different disorder from BED [157,158,159,160].

Since BED is now considered as proper diagnosis, there is a renew interest on the animal models used in order to uncover the neurobiological basis of BED. Herein we will review the most relevant features of some of these models.

### 4.1. Lessons Learned from Animal Models

Animal models can provide invaluable insight for neurobiological disorder research, especially when the aetiology is well characterised or when there are potentially genes associated. Current animal models of BED can only provide some few characteristics of the human disease. These models cannot reproduce all social context that influence human eating behaviour; neither some psychological aspects, such as sense of lack of self-control, blame or guilt. Moreover, there is not really a consensus about the criteria an animal model should fulfil [161]. This is major drawback considering the existence of strong differences with some rodent strains, e.g., obesity-prone vs. diet-resistant, that could be at the root of some discrepancies. In addition, one of the main features of BED is the much higher incidence in females and at specific stages of life as adolescence. The fact that the precise correlation between age of laboratory rats and human is still a subject of debate and the generalized use of male rats are further limiting factors. Despite this, these models have contributed to the understanding of the eating disorders, mainly trying to elucidate the neurobiological mechanisms implicated.

Many of the results obtained from animal models of eating disorders have been already discussed throughout the different epigraphy of this article. Likewise, more in detail reviews about this topic have been written over the last years [129,162,163,164,165,166,167,168,169,170]. Therefore, we will look briefly the plausible role of the genetic and environment background, and the relevance of different dietary macronutrients used in BED models that have not been discussed yet. Finally, we will highlight the state of the art of new drugs for the treatment of BED.

#### 4.1.1. Genetic Factors

Although the genetic study of BED is still in early stages and there are no enough genome-wide association studies (GWAS) focused exclusively on BED, preliminary evidences suggests that could there be predisposing risk factors [171,172,173,174]. In fact, there are good evidences that heritable factors make a significant contribution to the risk of developing eating disorders [175]. Patrono et al. showed that exposure to environmental conditions induces compulsion-like eating behaviour, depending on genetic background. Therefore, when they compared two mice inbred strains, C57BL/6J and DBA/2J, they observed that only DBA mice shift from motivated behaviour to compulsive eating behaviour. They hypothesised that this result could be explained as a genetic vulnerability (low accumbal D2 receptors availability observed in this study) [176]. This same year, the Bryant group has reported two genetic factors implicated in the development of BED in mice models. They observed that C57BL/6NJ but not C57BL/6J mice showed rapid and robust escalation in palatable food consumption and identified Cyfip2 (cytoplasmic FMR1-interacting protein 2) as a major genetic factor in preclinical BED; suggesting that could be associated with maladaptive feeding in humans. Based on the association of Cyfip1 with Prader-Willi syndrome, one hypothesis is that Cyfip1 polymorphisms also affect BED and hyperphagia [177]. At the same time, they showed Csnk1e (casein kinase 1 epsilon) deletion increased binge eating behaviours by enhancing opioid-induced locomotor activity [178]. Moreover, these authors have also reported a robust strain difference in BED (C57BL/6J versus DBA/2J strain) in accordance with previous results. Therefore, D2J showed escalation in consumption, conditioned place preference for the food-paired side and compulsive-like eating in an anxiety-provoking environment relative to B6J [179]. In this same context, rat strain differences in binge eating proneness have been examined. Results showed that the Sprague-Dawley female strain is particularly vulnerable to binge eating behaviours, while the Wistar female rat strain is particularly resistant to BED. Moreover, Sprague-Dawley males showed low risk for binge eating too. Taken together these results highlight the key role of sex and genetic backgrounds in the propensity to binge eating behaviours [180]. Despite of oestradiol reduces meal size and is associated with reduced binge frequency, women are more likely to suffer from BED. In a fat binge eating animal model it was observed that ovarian hormones, oestradiol and progesterone, have a tonic inhibitory effect on food intake, but the normal cyclic inhibitory effect on eating was disrupted during binge-type eating episodes. This observation suggests that these inhibitory effects of oestradiol could be compromised by binge-type consumption of large fatty meals [181]. Concurrently, other study has reported that bingeing also attenuated oestradiol tonic effects. While both tonic and cyclic inhibition of chow intake was maintained after cyclic treatment of ovariectomised rats with oestradiol alone or co-administrated with progesterone, neither tonic nor cyclic inhibition of binge-type consumption was observed once bingeing was fully established [182]. Both studies indicate that oestrogens are the primary ovarian hormones responsible for food intake and body weight regulation under binge-type conditions in rats. The oestrogen metabolite, 2-hydroxyestradiol (2OHE2), has been hypothesised may enhance bingeing in rodent models due to its possible interference with DA signalling. In fact, 2OHE2 may competitively inhibit degradation of DA and/or mimic or enhance D2 receptor actions [183]. Corwin’s group showed as chronic administration of 2OHE2 to ovariectomised female rats can exacerbate the induction of binge-type eating. Although they found that 2OHE2 attenuates the increase of food intake associated with ovariectomy, eating behaviour turns toward a binge-type pattern, when 2OHE2 is administrated before binge development and during all binge process. Therefore, this study suggests that chronic exposure to 2-hydroxyestradiol facilitates “learning to binge” [184]. The binge-eating proneness is also dependent on age. For both rat strains studied, Otsuka Long Evans Tokushima Fatty (OLETF) and lean control strain, Long Evans Tokushima Otsuka (LETO), the onset of binge eating behaviour was observed earlier in adolescents, with bigger binge size too. Higher impulsivity in adolescents could explain their increased vulnerability to BED and overeating. Furthermore, OLETF rats at both ages ate more than LETO rats. This is in accordance with the co-morbidity of BED and obesity reported in humans [185]. However, not always the binge eating behaviour is dependent on susceptibility to obesity, as animal models have confirmed [186,187]. The development of aberrant eating behaviour during adolescence has been also studied in other animal models (Sprague-Dawley strain). After postnatal treatment with tricyclic antidepressant clomipramine, female rats showed more vulnerability to exhibit binge-like eating behaviour during adolescence [188]. Sex differences in coping strategies with anxiety have been described clinically. Female strategy, more consistent with harm avoidance, has been associated with BED [189]. On the other hand, it has been proposed that cognitive deficits in executive function, inhibitory control, attention, and mental flexibility may play a role in development and sustaining of binge eating behaviour. When studying a limited access rat model of binge-like behaviour, Chawla et al. found aberrant gene expression of brain derived neurotrophic factor (BDNF) and tropomyosin receptor kinase B (TRKB) in the hippocampus (HPC)-prefrontal cortex (PFC) pathway. Moreover, they observed reductions in the expression of insulin receptor in the CA3 region of the hippocampus, up-regulation of serotonin-2C receptors in the orbitoprefrontal cortex and decreased dopamine receptor 2 expression in the nucleus accumbens (NAc). Altered expression of genes in the neural pathway HPC-PFC-NAc could be explained as consequence of engaging in the binge behaviour. Authors have also speculated that animals binge prone could have cognitive deficits and these contribute to their vulnerability [190]. Similar results were recently found in BED patients. Based on GWAS it has been possible to identify genes involved in neuropeptide/neurotrophic pathways including neurotensin, GLP1 and BDNF-TRKB signalling as potential therapeutic targets [191]. On the other hand, evidence of epigenetic modification affecting the N/OFQ (Nociceptin/Orphanin FQ) and corticotropin-releasing factor (CRF) systems in response to food restriction and stress exposure has been demonstrated. These mechanisms could be designed to maximize the possibility of survival in the presence of food restrictions, but in a society where nutrients are easily available, alteration of N/OFQ and CRF mechanisms may contribute to BED development [192].

#### 4.1.2. Environmental Factors

Among the many environmental factors that can influence eating disorders together with the high palatable food availability, the high incidence of stress in western societies is probably one of the most relevant [169,193]. It is well documented with different animal models that stress can influence feeding behaviour [193,194,195,196,197,198] and increase the susceptibility to developing eating disorders [167]. Most of the animal models have focused the investigation on the regulation of the stress neurohormone corticotropin-releasing factor (CRF) system. Calvez et al. have found that stress differentially regulates brain expression of CRF in binge-like eating prone (BEP) and resistant (BER) female rats. Therefore, in response to stress, the BER rats significantly enhanced expression of CRF mRNA in the paraventricular nucleus of the hypothalamus and strongly increased the plasma corticosterone levels. Conversely, the BEP rats did not displayed stress-induced activation of the HPA axis, but they demonstrated high CRF mRNA expression in the oval and anteroventral bed nucleus of the stria terminalis (BNST), an important region for the motivated behaviour, in response to stress [199]. Moreover, BEP rats showed different behavioural and hormonal responses to stress. They showed higher motivation for palatable food and perceived hedonic value [200]. Function of BNST was also assessed in other models. Indeed, frustration stress manipulation increased BNST neuronal activity and CRF receptors of BNST seem to have a critical role in stress-induced binge eating disorder [201]. The role of other extra-hypothalamic CRF1 receptors, as those in central amygdala, has been also implicated in development of binge eating [202]. The early emotional environment also impacts on eating behaviour. Early attachment experiences increase the risk of eating disorders. In fact, the impact of perinatal programming on the mechanisms regulating body weight homeostasis has been fully studied [203]. This same year, an interesting work has shown that late gestation prenatal stress rewires neural circuits in female mice, leading to binge-like behaviour [204]. Authors reported that overexpression of maternal CRF, during late gestation affects the hypothalamus of female offspring, altering the expression levels of genes responsible for DNA methylation and predisposes adolescent female offspring to BE-like phenotype. Offspring with BE-like behaviour presented hypomethylation of hypothalamic miR-1a and downstream dysregulation of the melanocortin system through Pax7/Pax3. Finally, authors showed that although stress-related epigenetic predisposition can increase the vulnerability to BED, it can be prevented from being triggered with a methyl balanced diet during adolescence.

#### 4.1.3. Food Models: Sugar Model, Fat Model, Sweet-Fat Model

The sugar bingeing model was proposed by Avena and collaborators [158,205]. This model uses sucrose, glucose and saccharin in different liquid solutions. After some days under this food animals modify the feeding behaviour and it is possible to induce neurochemical changes in the brain. Even, this model increases DA in NAc [206] and cessation of sweet food availability induces withdrawal-like behaviour [93,207]. Following withdrawal from ad libitum access to sugar diet, animals also showed depressive-like behaviour [208,209]. However, in a Wistar rat model of binge eating based on daily 10-min access to a sweet fat diet, there was no increased anxiety-like behaviour after diet withdrawal. In this same model, it was observed that pre-treatment with the cannabinoid type 1 receptor antagonist SR147778 dose-dependently reduced binge-like intake, while binge-like intake was unaffected by pre-treatment with the corticotropin-releasing factor type 1 receptor antagonist R121919 [210]. Sugar model has also been reported to increase the cross-sensitization with other substances [129,205]. Recently, it was observed as binge-like sucrose consumption reduces the dendritic length and complexity of principal neurons in basolateral amygdala (BLA) in an adolescent rat model. These maladaptive changes observed in dendritic architecture of BLA principal neurons, particularly on apical dendrites, show the importance of the BLA in encoding emotional salience [211]. Furthermore, sugar models have provided evidence of the relevance of dietary restraint as an antecedent to sugar binges, in concordance with human studies [212] and can reproduce one human feature of binge eating known as “eating when not physically hungry” [213]. The studies suggest that, as with sugar models, a similar addiction-like state may emerge with fat intake. Insight in this issue was obtained with the limited access model proposed by Corwin [214,215]. In this experimental paradigm, the rats are given sporadic (generally 3 times per week, binge group) or time-limited (daily access control group) access to palatable food, in addition to the continuously available chow. The palatable food typically is a bowl of pure vegetable shortening, but other palatable food can also be tested including sucrose solutions, various concentrations of fat presented as solid emulsions, high-fat diets, and fat/sucrose mixtures. In terms of neurochemical changes, it has been reported as binge eating of fat affects DA signalling in the accumbens and γ-aminobutyric acid (GABA_B_) receptors. In fact, licking of 100% corn oil increases DA and its metabolites in the NAc, similar to that seen in sugar-bingeing animals [216]. On the other hand, peripheral administration of the D2-like antagonist raclopride stimulated fat intake in intermittent-access rats and had no effect in daily-access rats. These results further implicate D2 receptors in the consumption of fatty food, but also indicate as well as uncover the role of differential pre- and post-synaptic D2 signalling under binge and control conditions [215,217]. In this same study, Corwin and colleagues have reported the role of GABA_B_ receptors in fat binge eating models was studied. GABA_B_ agonist baclofen reduced intake of shortening, as well as high-fat solid emulsions, in rats with both daily and sporadic brief access at doses that stimulated or had no effect on chow intake [217,218]. However, baclofen did not have effect on intake of sugar solutions or when sugar concentration was high [186,219,220]. Corwin’s model as well, has shown an increase in progressive-ratio responding in rats under binge eating fat, suggesting enhanced motivation [215]. Moreover, a history of fat bingeing may predispose to exhibit more robust addiction like behaviour to other abuse substances [221]. In contrast to sugar models, animals under fat bingeing models did not display withdrawal-associated symptoms [222], and even after several weeks fat-fed animals did not show significant changes in body weight, supporting the idea that BED and obesity are two different pathologies [129]. It has been proposed, that lack of opiate-like withdrawal signs in fat-bingeing rats may be caused by fat-induced endogenous galanin activation, which can inhibit the relevant opioid effects [163]. Finally, many studies have used a sweet-fat model (“cafeteria-diet”), which is probably the model most similar to the huge availability and diversity of foods that we have nowadays. According to sugar model, sweet-fat food also induced a downregulation of DA mesolimbic pathway [89]. Moreover, after exposure to cafeteria diet, following acute withdrawal animals showed increase in locomotor activity, stress and anxiety-related behaviour [207,208,223,224].

#### 4.1.4. Neuropharmacology of Binge-Eating Behaviour

The treatment of binge eating disorder is challenging and require a big-picture treatment plan to meet the individual needs; combination of psychotherapy and medication is usually the best treatment strategy [130]. Oral lisdexamfetamine dimesylate (LDX), a prodrug of dextroamfetamine, is currently the only drug to be approved in the USA for the treatment of moderate to severe BED in adult patients [225]. When given acutely in a female rat BED model, LDX attenuated binge-eating of chocolate without influencing the consumption of normal chow or affecting the bodyweight of the animals [226] and reduced impulsiveness and perseverative behaviour of binge-eating rats in a novel food reward/punished responding conflict model [227,228]. Pharmacological characterisation indicated that LDX attenuates binge-eating in part by indirect activation of α1-adrenergic and possibly also dopamine D1 receptors in the CNS, but α2-adrenergic and D2 receptors are not involved [226]. Other novel pharmacological treatment approaches also give us additional information regarding the underlying neuro-pathways implicated in BED. For instance, the D2 receptor antagonist raclopride reduced sucrose intake [220]. Methylphenidate (MPH), which inhibits the monoamine uptake transporters for DA and norepinephrine, also reduces sucrose bingeing episodes in animal models. Concomitant with this, MPH treatment leads to increased DA transporter and D2 receptor binding in the NAc shell [229]. GS 455534, an aldehyde dehydrogenase-2 inhibitor that reduces DA synthesis has also been shown to selectively reduce binge consumption of sugar. Moreover, GS 455534 is associated with an attenuation of nucleus accumbens dopamine levels in sugar-bingeing rats model [230]. In addition, it was observed that monoamine stabilizer (−)-OSU6162, which restores striatal dopaminergic dysfunction, could be a novel BED treatment in rodent models since reduces both binge-like eating and cue-controlled food seeking in rats [231]. Selective serotonin reuptake inhibitors (SSRIs) have been also studied. Fluoxetine non-selectively reduced the intake of normal food and highly palatable food in a model of binge-eating that included cyclic caloric restriction and stress [232]. Moreover, it has been reported that fluoxetine and d-fenfluramine (a serotonin releasing agent) reduced binge-eating in mice on an intermittent high fat diet by enhancing midbrain dopamine neuronal activity [103]. Sibutramine, a monoamine reuptake inhibitor (MRI), removed from the worldwide market because of an increased risk of myocardial infarction and stroke, has been also studied in BED animal models [226,232], where reduced the binge eating behaviours. On the other hand, selective 5-HT2C receptor agonist lorcaserin, developed and approved as an anti-obesity agent, was recently shown to attenuate binge-eating in mice fed a high fat diet by stimulating dopamine neuronal activity [103]. In a recent study, it has been reported for the first time that trace amine-associated receptor-1(TAAR1) may represent a novel target for the treatment of BED. RO5256390, a TAAR1 partial agonist, completely blocked compulsive-like eating and conditioned rewarding properties of palatable food when studied in a rat model with limited access to a highly palatable sugar diet [233].

As we have already mentioned previously, the opioid system plays a key role in binge behaviours. In fact, opioid receptor antagonists, including naltrexone, nalmefene and GSK1521498 decreased both normal food intake and compulsive or excessive eating in several different binge-eating models in rats [55,226,234]. However, despite the positive pre-clinical evidences, placebo-controlled clinical studies in binge-eating disorder with opioid receptor antagonists have been disappointing [130].

The orexin system plays a role in eating disorders characterised by compulsive binge-type episodes, such as BED. For instance, in two different rat compulsive binge-eating models orexin receptor 1 (OXR1) antagonist SB-334867 and GSK1059865 specially reduced palatable versus normal food intake [226,235]. However, JNJ-10397049 a selective orexin receptor 2 (OXR2) antagonist, failed to affect the intake of palatable food and the dual OX1/OX2 receptor antagonist SB-649868 selectively reduced binge-eating of highly palatable food but did not affect the chow food pellet intake [235]. In this same context, it was observed that SB-334867, a selective OXR1 antagonist, decreased the binge-like consumption behaviour in an ad-lib feeding animal model [236].

The combination of pharmacological agents acting on different neural pathways may also provide novel therapy for BED. Modulation of GABAergic neural systems also represents a possible approach for BED treatment. Baclofen, a GABA_B_ agonist, attenuated the intake of highly palatable food in several different animal models of binge-eating [219,220,226,237]. Recent studies have suggested Nociceptin/Orphanin FQ (N/OFQ) system, a functional antagonist of corticotrophin-releasing factor, as therapeutic target either obesity or BED. In fact, intracerebroventricular injections of N/OFQ at low doses significantly reduced BE in rats [238]. On the other hand, nociceptin receptor antagonist LY2940094 [239] and SB 612111 [240] inhibits excessive feeding behaviour in rodents. Interestingly, a novel drug for the treatment of BED, may be BD-1063, a selective sigma1 receptor antagonist. Although the selectivity of this compound has not been well characterised, BD-1063 blocked compulsive binge-eating in rats trained to obtain a sugary or highly palatable diet [241].

## 5. Conclusions and Future Perspectives

**Figure 1:**
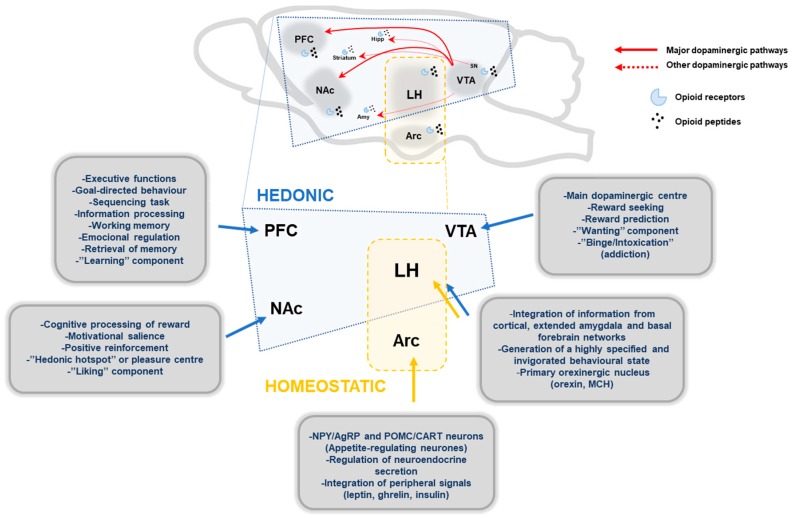
Schematic representation of main brain structures implicated in hedonic and homeostatic food intake regulation. Main dopaminergic pathways, mesolimbic and mesocortical pathway, are represented with red lines, and other minor dopaminergic connections with broken red lines. Endogenous opioids peptides modulate the dopaminergic pathways through opioid receptors (µ, κ, δ). Hedonic pathways: PFC, prefrontal cortex; NAc, nucleus accumbens; VTA, ventral tegmentum area; LH, lateral hypothalamus; Amy, Amygdala; Hipp, Hippocampus; SN, substantia nigra. Homeostatic pathways: Arc, arcuate nucleus; MCH, melanin concentrating-hormone.

This review briefly presents the latest knowledge regarding the neurobiology of non-homeostatic pathways implicated in food intake regulation. Throughout the review we have tried to discuss the most relevant aspects that can help us to understand the dysregulation of brain reward systems in eating disorders, by focusing mainly on BED. However, me must be aware that in most instances it is far from clear whether the changes are related to the disease, consequence of altered homeostasis or a premorbid trait. During recent years, research in animal models of compulsive overeating has provided us essential knowledge to understand the neurobiology mechanisms underlying eating disorders, but there are still many features waiting to be elucidated. The main brain structures implicated are shown schematically in Figure 1. Among the mechanisms involved the role of dopamine appears clear-cut. Recent neuronal mapping using single-cell RNA-seq. have shown the existence of a higher diversity of neurones than originally expected with up to 62 neuronal subtypes producing glutamatergic, dopaminergic or GABAergic markers for synaptic neurotransmission having been identified. The challenge now is to associate the different subsets of neurones to specific neurobiological process and their alterations to specific disease-related process. After BED has been recognised as a proper psychiatric disorder, clinical and basic research in BED is in an exciting phase among other reasons because of the availability of new neuroimaging tools that allowed us to correlate basic neurobiological aspects to functionality in the human brain. Development of well-characterised models and new tools such as chemogenetics and optogenetics should provide us with a much better mechanistic insight and possible therapeutic targets for BED. Finally, we should not forget that any functional aspect of the brain is based on the complex interaction among many different neuronal pathways. It is expected that as soon as larger knowledge is gather from the connectome project [242] we will be in a much better position to tackle questions related to complex diseases such as BED and how nutrient intake, in terms of total amount and schedules, can influence cognitive process.
